# Safety of combined exercise and spinal cord stimulation therapy in persistent spinal pain syndrome: a case report

**DOI:** 10.3389/fpain.2026.1760565

**Published:** 2026-04-10

**Authors:** J. Vicente-Mampel, D. Sánchez-Poveda, M. Martínez-Soler, F. Hernández-Zaballos, J. Ferrer-Torregrosa, F. J. Sanchez-Montero

**Affiliations:** 1 Department of Physiotherapy, School of Medicine and Health Science, Catholic University of Valencia, Torrent, Valencia, Spain; 2 Anesthesiology Service, Pain Unit, Complejo Asistencial Universitario de Salamanca (CAUSA), Salamanca, Spain; 3 Department of Podiatry, School of Medicine and Health Science, Catholic University of Valencia, Torrent, Valencia, Spain

**Keywords:** chronic pain, exercise, failed back surgery syndrome, persistent spinal pain syndrome, spinal cord stimulation

## Abstract

A 36-year-old man with a history of lumbar disc herniation and laminectomy in 2022 presented with recurrent pain, despite initial improvement. His symptoms, likely attributable to postsurgical fibrosis and residual disc protrusion, were managed with various treatments, including pharmacological therapy, epidural steroid injection, and epidurolysis, albeit with limited success. Following a multidisciplinary evaluation, SCS was proposed, and the patient consented to the procedure. Diagnostic imaging revealed postsurgical changes and nerve root involvement at the L5-S1 level. The patient commenced a structured exercise program in conjunction with SCS, aimed at enhancing spinal stability and function, with positive outcomes anticipated through continued intervention. The current findings suggest that exercise, when following a standardised protocol, carries a minimal risk of electrode migration. However, further research is required to validate the reliability of these radiographic measurements. Future studies should focus on larger sample sizes and examine the combined effects of SCS and individualized exercise interventions. This could aid in the development of more effective and comprehensive treatment protocols for PSPS-T2 patients.

## 1 Introduction

The management of persistent spinal pain syndrome (PSPS) necessitates a multidisciplinary approach. This may include using medicines, physical therapy, mental health support, and certain medical procedures. It is important to set realistic goals and educate the patient (1). The classification comprises two subtypes: PSPS-type 1, which occurs in the absence of prior spinal surgery, and PSPS-type 2, characterized by chronic persistent pain following spinal surgery (2). Various therapeutic strategies have been employed by pain specialists for patients experiencing failed back surgery syndrome. These strategies include exercise, physical therapy, and cognitive-behavioral rehabilitation; pharmacological management; interventional procedures such as epidural injections; neuromodulation via spinal cord stimulation (SCS); and, in certain instances, reoperation. Although conventional interventional techniques frequently yield only temporary relief, spinal cord stimulation has demonstrated sustained benefits at medium-term follow-up (up to 2–3 years) (3). Spinal cord stimulation (SCS) has been demonstrated to be an effective intervention for chronic, intractable neuropathic limb pain and may also offer benefits to carefully selected individuals experiencing axial pain (4).

Initial randomized controlled trials have established the efficacy of SCS in patients with failed back surgery syndrome, demonstrating outcomes that are comparable to or surpass those of repeat surgical interventions and conventional medical management (5, 6). The initial results of the meta-analysis revealed that, after a period of 12 months, 68% of patients experienced a reduction in leg pain, 63% reported a decrease in back pain, and 73% noted a reduction in overall pain. At the 24-month mark, 63% of patients continued to report diminished leg pain, 59% experienced reduced back pain, and 71% maintained lower levels of general pain (7). In addition to its clinical effectiveness, SCS has also been recognized as a cost-effective treatment that can reduce the economic burden associated with PSPS (8).The management of PSPS highlights the importance of adopting a comprehensive multidisciplinary approach. This patient-focused method involves customizing treatment plans to address the specific needs and situations of each person (9). The integration of SCS with adjunctive therapies is crucial, as SCS alone frequently fails to achieve optimal functional outcomes (10). Objective assessment tools are instrumental in evaluating neuropathic pain, while functional outcomes—including the ability to return to work—are optimized through a personalized multidisciplinary rehabilitation program (11). Evidence suggests that only 9.5%–14% of patients with SCS can return to work without additional therapy, highlighting the necessity for combined interventions and the development of appropriate functional rating scales (12).

The integration of the analgesic effects of SCS with those derived from exercise may hold particular significance for patients with PSPS. The combination of these treatments has the potential to enhance self-management and modify patients’ beliefs. For example, catastrophizing has a substantial impact on how patients with PSPS/T2 perceive pain and experience disability during SCS (13). Although combining both treatments could be considered a key approach to improving outcomes in patients with PSPS (14), a premature return to strenuous activity is considered a risk factor for lead migration (15).

Recently, different anchoring methods have been used successfully in clinics. They help reduce the number of lead movements and breaks. However, the time needed for fibrous tissue to fully encapsulate is still unknown (16). As per the guidelines established by the Neurostimulation Appropriateness Consensus Committee (NACC), it is essential for patients to engage in a regimen of progressively increasing physical activity following SCS implantation to effectively manage chronic pain. Initially, patients are advised to refrain from activities such as bending, twisting, stretching, and heavy lifting. After a period of two weeks, they may gradually transition to light activities, such as walking. This strategy is designed to mitigate the risk of complications, including lead migration or fracture, which are more commonly associated with cervical placements. Understanding the healing timeline of epidural electrodes, estimated between six and 12 weeks, aids in determining safe activity resumption (17).

We report the first case of a patient combining a protocol for exercises adapted to the NACC guidelines 12 days post-implant (18). This case study examines the uncommon instance of an absence of lead migration in a patient diagnosed with PSPS-T2, who immediately integrated the exercise protocol with SCS following the intervention. The evaluation concentrated on the short-term risk period, as this is when lead migration is most probable due to the lack of fibrotic encapsulation around the electrode. This initial phase is crucial for assessing stability, given that the electrode has not yet been secured by the tissue healing process. The aim of this case report was to evaluate the short-term safety of an early structured exercise protocol initiated 12 days after SCS implantation in a patient with PSPS-T2, focusing on the risk of lead migration.

## 2 Case report

### 2.1 Patient information

A 36-year-old male with no notable medical history underwent laminectomy for lumbar disc herniation in 2022. The initial postoperative recovery was favourable, with symptom improvement observed within the first 15 days. However, the patient subsequently experienced a recurrence of pain, leading to a referral for further evaluation in April 2023. The pain appeared to have originated following the initial disc herniation, which ultimately necessitated surgical intervention. The current symptoms were likely associated with postsurgical fibrosis and residual disc protrusion at the L5-S1 level. Initially, the patient received standard pharmacological treatment aimed at managing neuropathic pain. Despite adherence to medication and conservative measures, his symptoms persisted, significantly affecting his quality of life. In November 2023, an epidural steroid injection was administered as part of an interventional pain management strategy. However, this approach did not result in meaningful or clinically relevant improvements. Given the refractory nature of the pain, the patient subsequently underwent epidurolysis the following day. Following the procedure, the patient experienced a slight reduction in pain intensity, which lasted for approximately 15 days before returning to baseline levels. Considering the limited and transient response to prior treatments and after a thorough multidisciplinary assessment, including evaluation by members of the pain management unit and a formal psychological evaluation, the possibility of SCS was presented to the patient as a subsequent treatment option. The rationale, procedure, risks, and expected outcomes associated with neuromodulation were also explained. The patient demonstrated adequate comprehension of the information provided and provided informed consent to undergo a spinal cord stimulation trial.

### 2.2 Clinical findings

The patient exhibited low back pain, aggravated by both flexion and extension of the lumbar spine. Tenderness was observed over the lumbar spinous processes, and pain was provoked upon palpation of the lumbar facet joints. The patient also reported neuropathic pain radiating through the left lower limb, extending to the lateral malleolus. Neurological assessments revealed positive Lasègue and Bragard signs at 20° on the left side. The pain was characterized as mixed, with a predominant neuropathic component, as evidenced by a DN4 score of 6 out of 10. Functional evaluation indicated a high level of disability, with an Oswestry Disability Index score of 65%, signifying severe impairment. These findings suggest a clinical presentation of mixed lumbar pain, with a significant neuropathic component likely attributable to nerve root involvement. Further diagnostic evaluation and an individualized treatment strategy are necessary to address both the nociceptive and neuropathic components of the condition.

### 2.3 Timeline

**Figure 1:**
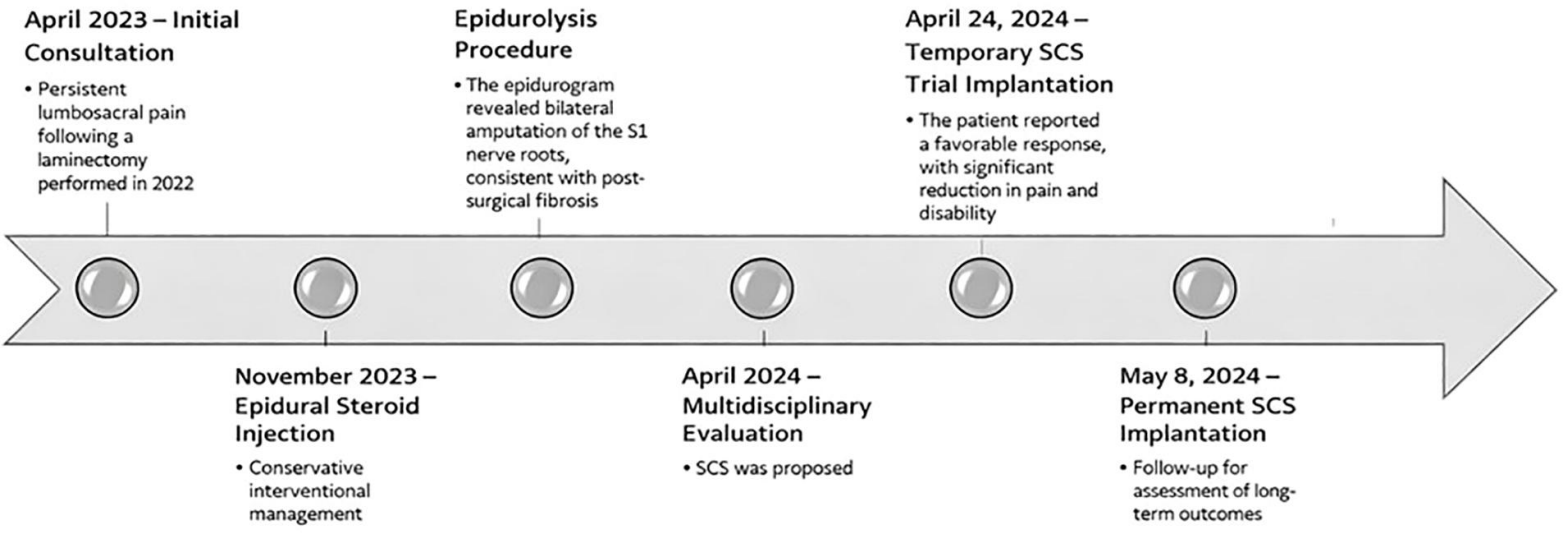
Timeline of clinical management, including initial consultation, epidural steroid injection, epidurolysis, multidisciplinary evaluation, and subsequent temporary and SCS.

The patient's clinical course is outlined in the timeline provided below (Figure 1).

### 2.4 Diagnostic assessment

In 2023, lumbar magnetic resonance imaging (MRI) identified postsurgical alterations from L4 to S1, consistent with left-sided laminectomy. At the L4-L5 level, degenerative disc disease with mild annular protrusion was noted without definitive evidence of nerve root compression. At L5-S1, a persistent central posterior disc protrusion was observed, which obliterated the epidural fat and made contact with the origin of both S1 nerve roots. The left S1 root demonstrated a loss of periradicular fat signal on T1-weighted sequences, indicative of post-treatment fibrosis. Additionally, during the epidurolysis procedure, an epidurogram revealed evidence of bilateral S1 nerve root amputation, suggesting significant postsurgical fibrosis and adhesion formation.

### 2.5 Therapeutic intervention

#### 2.5.1 Exercise

Scientific summary in table format of the experimental intervention described. This table outlines the core stability and control motor training program with neurostimulation in a clear and structured way (Table 1) (18). A lumbo-pelvic core stability training program that incorporated motor control exercises targeting the lumbopelvic region, combined with neurostimulation treatment. This intervention plan was designed in accordance with the principles established by Falla et al. (19). The exercises in each phase were specifically structured to limit the degree of flexion/extension and lumbar traction. Participants completed two weekly sessions, each lasting 60 min over eight weeks, for a total of 24 sessions. Parameters such as series, repetitions, intensity, duration, break, and effort were extracted directly from the published article to ensure reproducibility and adherence to established therapeutic guidelines (18).

**Table 1:** Structured presentation of the training program combining core stability, motor control exercises, and spinal cord stimulation.

Phase	Duration	Objectives & Focus	Exercise Characteristics
Phase 1 Muscle Activation	1–15	Achieve voluntary neuromuscular control	- 4 sessions - No spine flexion/extension >65° - Techniques: rib breathing, forced exhalation - Activation of: internal obliques, multifidus, transversus abdominis - Use of ultrasound feedback
Phase 2A Posture/Alignment	16–37	Strengthen deep spinal stabilizers	- 6 sessions over 21 days - Flexion: up to 65°, Extension: up to 85° - Home exercises with monitoring - 1–3 series, 8–15 reps, isometric holds (5–10 s) - 30 s rest between series, 2–3 min between exercises
Phase 2B Posture/Alignment	38–60	Phase 2A, with added voluntary spinal traction for enhanced postural control	- Exercise protocol remains the same as Phase 2A
Phase 2C Posture/Alignment	61–90	Expand ROM and reinforce control under isometric conditions	- Flexion: up to 90°, Extension: up to 85° - Exercises remain isometric - Progression individualized - 1–3 series, 8–15 reps, 5–10 s contractions - 30 s rest between series, 2–3 min between exercises
Phase 3 Movement Strategies	90+	Integrate full movement into functional activities; ensure electrode stability	- Flexion: up to 155°, Extension: up to 115° - Use of concentric and eccentric contractions - Functional movement emphasis

#### 2.5.2 Spinal cord stimulation procedure

SCS is a therapeutic approach involving the use of an implantable pulse generator designed to enhance therapeutic outcomes through various stimulation algorithms and parameters (20). This therapy targets distal areas, such as the dorsal root ganglion, offering potentially greater anatomical specificity. Furthermore, subthreshold stimulation, which employs high-frequency or burst energy delivery, can reduce or eliminate unwanted paraesthesia and off-target sensations. Research has shown that subthreshold stimulation at higher frequencies or using different stimulation patterns can provide pain relief comparable to or even more effective than traditional SCS (21). The procedure involved placing two octopolar electrodes into the epidural space, positioned beneath the posterior region of the spinal cord, specifically posterior to the spinal cord's dorsal horn. The electrodes were positioned between the T8 and T11 vertebrae, corresponding to the area with the highest synaptic activity in the spinothalamic tracts, responsible for processing pain signals from the legs and lumbar region (22). The cylindrical percutaneous leads employed were secured to the fascia utilizing the Inyex system®. Lead migration represents the most prevalent hardware-related complication associated with spinal cord stimulation. However, recent systematic reviews and clinical studies indicate that clinically significant migration occurs in approximately 10% of patients who have undergone implantation (23, 24). The procedure employed in this study was the Differential Target Multiplexed (DTM) spinal cord stimulation protocol, delivered using the Inceptiv™ system. This advanced neuromodulation approach differs from conventional SCS by targeting both neuronal and glial cell activity to achieve a more comprehensive modulation of the spinal cord microenvironment for chronic pain management.

### 2.6 Follow-up and outcomes

#### 2.6.1 Radiological assessment

**Figure 2:**
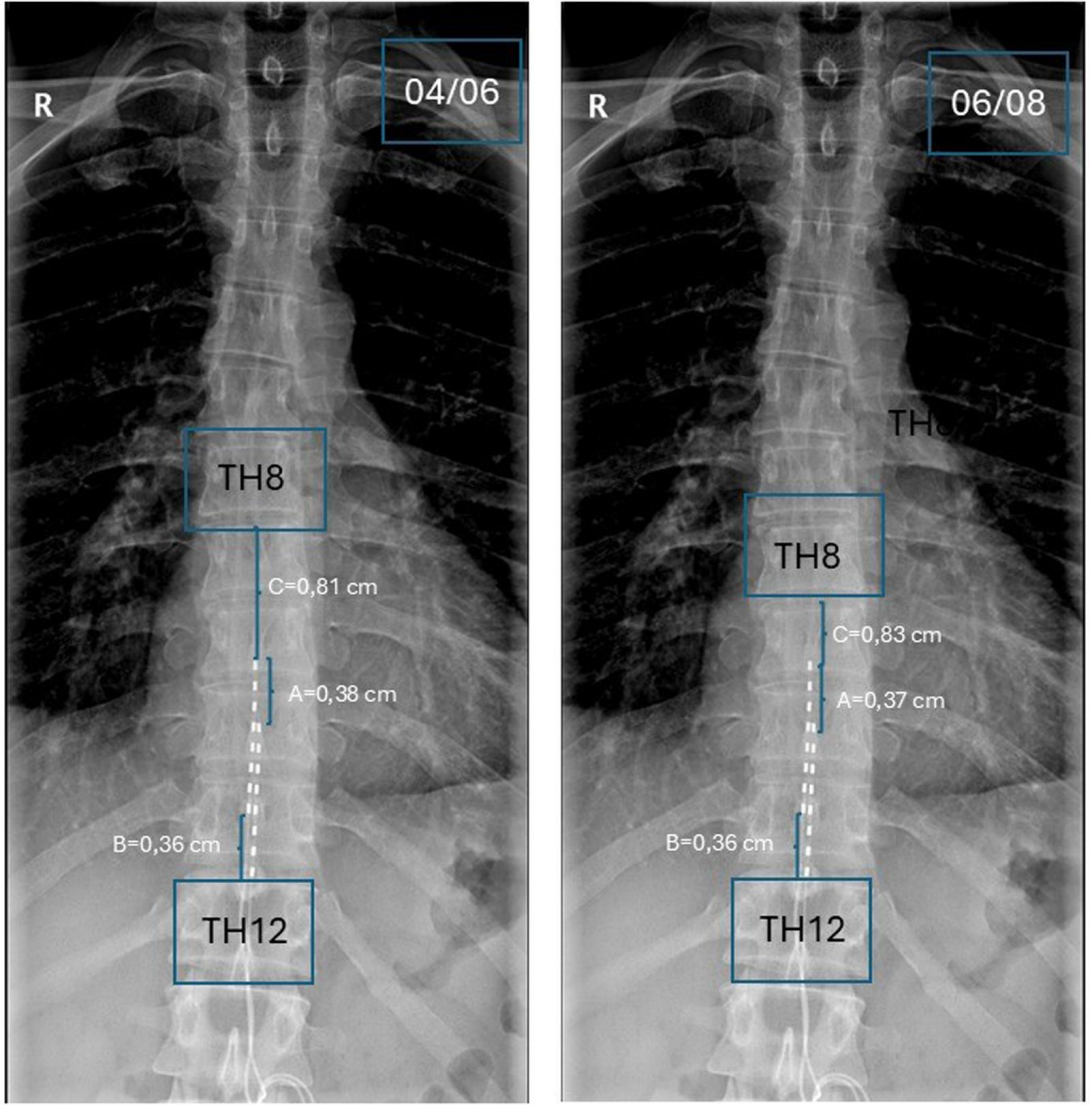
Comparative anteroposterior thoracic radiographs obtained on 04/06 (left) and 06/08 (right), showing TH8–TH12 levels and linear measurements (A, B, C) for assessment of vertebral alignment, with no significant interval change.

Anteroposterior radiographs of the thoracic region were employed to assess potential electrode migration through image analysis. Measurements were conducted using ImageJ, a processing and analysis software developed by the National Institutes of Health. ImageJ enables standardized radiographic measurements by calculating distances and spatial relationships in digital images, thereby ensuring precision and reliability in quantitative image analysis. To further standardize the measurements and account for operator-dependent variability in radiograph acquisition, the total length from the upper endplate of the T12 vertebral body to the lower endplate of the T8 vertebra was calculated (Vector A). From this reference point, the distance from the upper electrode to the lower endplate of the T8 vertebra was measured (Vector B). Similarly, Vector C was defined as the distance from the lower electrode to the lower endplate of the T8 vertebra. All measurements were recorded in centimeters. Radiographic examinations were performed following electrode placement and again two months post-intervention. The percentage change was subsequently assessed after the definitive implantation of the SCS device. The findings of this evaluation are illustrated in Figure 2.

#### 2.6.2 Short-term clinical outcomes

The following table provides a summary of the observed changes across key clinical measures, indicating improvements in most domains, except for a slight increase in pain catastrophizing (Table 2).

**Table 2:** Summary of short-term changes in key clinical outcomes following intervention.

Variable	Baseline	2 Months	Clinical Change
Visual Analog Scale (Pain Perception)	8	6	−2
SF-12 Physical Component Score	17.68	20.93	+3.25
SF-12 Mental Component Score	52.95	50.95	−2.00
Central Sensitization Inventory	27	18	−9
Oswestry Low Back Pain Disability Questionnaire	48	13	−35
Pain Self-Efficacy Scale	44.16	61.15	+16.99
Pain Catastrophizing Scale	28	33	+5

### 2.7 Adherence and monitoring

Throughout the study, treatment was tailored to individual needs and closely monitored. Adherence to the rehabilitation protocol was assessed using both objective and clinical methods. Attendance at sessions was meticulously recorded, and healthcare providers conducted routine evaluations to assess patient engagement and compliance. To enhance adherence and minimize the risk of adverse events, personalized training sessions were conducted at the onset of the intervention to ensure participants fully comprehended the therapeutic plan and could safely execute all prescribed activities. No adverse events were observed or reported during the follow-up period.

## 3 Discussion

PSPS-T2 affects a broad population and is influenced by neuroplastic mechanisms as well as biological, psychological, and social factors (25). In response to this complex clinical presentation, advanced therapeutic approaches—particularly neuromodulatory strategies—have shown potential to promote muscle recovery during rehabilitation (26). Within this context, the immediate combination of the two neuromodulation-based treatments may be interpreted as a multidisciplinary therapeutic approach (27), with combined modalities potentially yielding superior outcomes in the short- and medium-term (28). Patient-centred approaches have recently been recognized as essential in the management of chronic pain (29). In this regard, exercise-based interventions extend beyond biomechanical correction and function as therapeutic strategies that promote self-management (30) and encourage the development of more adaptive biopsychosocial beliefs (31). However, current clinical recommendations following spinal cord stimulation (SCS) frequently advise avoiding strenuous physical activity during the early postoperative period.

Although growing evidence supports the potential benefits of combining SCS with exercise, and a randomized controlled trial protocol has already been developed and published (32), there is a paucity of outcome data evaluating the effects of this combined approach. Most existing studies have focused on each intervention in isolation, with limited research exploring their synergistic potential when applied concurrently. In contrast, combined neuromodulation strategies have been more extensively investigated in other clinical contexts, based on the hypothesis that simultaneous interventions may enhance therapeutic effects (33). It is also important to recognise that the term “exercise” encompasses a wide range of modalities, some of which may be safe and beneficial for individuals with chronic spinal pain. Numerous exercise interventions—including aerobic training, resistance exercises, directional movements, aquatic therapy, Pilates, yoga, core stabilization, and motor control exercises—have been investigated for their effectiveness in improving outcomes in patients with spinal pain (33).

The exercise protocol implemented in this study was specifically designed using controlled degrees of lumbar flexion, extension, and rotation. This approach is based on the premise that lumbar stabilization and motor control exercises can be tailored according to the mechanical stress imposed on neural tissues during spinal dysfunction. Movements performed at end-range positions may increase neural tension and potentially elevate the risk of lead migration, particularly when the electrode is located in the epidural space and the surrounding tissue has not yet developed sufficient fibrotic encapsulation. Therefore, graded progression was implemented to enhance neuromechanical safety while promoting functional recovery. The findings from the present case suggest that exercises performed according to this standardized protocol may represent a safe approach with a minimal risk of electrode migration in the short term. However, the 60-day follow-up period represents a limitation of this study. Longer follow-up periods, ideally ranging from 6 to 12 months, are required to confirm the long-term safety and effectiveness of the intervention, particularly considering that epidural lead healing may extend up to approximately 12 weeks. Studies with extended follow-up are currently underway to further explore this issue.

Several additional methodological limitations should also be acknowledged. Although radiographic evaluation was standardized using ImageJ software to quantify lead displacement as a percentage of distance, the reliability of these measurements remains limited. Radiographic methods for assessing lead migration are inherently subject to variability and lack full reproducibility, highlighting the need for standardized evaluation protocols. Nevertheless, the use of ImageJ represents a novel quantitative approach in the context of SCS for PSPS-T2, with the potential to enhance measurement precision. However, its clinical applicability should be validated in studies with larger sample sizes and longer follow-up periods. Moreover, several factors may influence electrode stability and the outcomes of exercise interventions following SCS implantation. These include the type and technique of lead anchoring; variability in surgical implantation methods; patient characteristics, such as body weight, musculature, and spinal anatomy; baseline physical activity levels prior to SCS implantation; and patient adherence to the prescribed exercise program. Although these variables were not specifically quantified in this case report, acknowledging their potential influence highlights the complexity of post-SCS rehabilitation and the importance of individualized patient management.

In conclusion, these methodological considerations should be addressed in future research to strengthen the validity of the findings and support more robust conclusions. Further studies with rigorous designs and broader patient populations are necessary to confirm the safety and efficacy of structured exercise interventions after SCS implantation.

### 3.1 Patient perspective

Initially, the patient expressed skepticism regarding the efficacy of the exercise program, voicing doubts about its potential to provide the anticipated relief and benefits. However, as the program progressed, particularly by the fourth week, the patient became more at ease with the exercises and gradually noted improvements in comfort and mobility. Over time, the patient's perspective evolved, and by the program's conclusion, they reported genuine satisfaction with the outcomes. The patient emphasized that the exercise intervention significantly enhanced their overall well-being and acknowledged its positive impact on managing their condition. This shift in outlook underscores the patient's growing confidence in the therapeutic value of the exercises and highlights the importance of consistent participation in rehabilitation programs to achieve long-term improvement. The findings from the current patient suggest that when conducted in accordance with a standardized protocol, exercise may present a minimal risk for electrode migration. Nonetheless, the limitations of this study must be acknowledged, particularly concerning the reliability of radiographic measurements, which require further validation through reproducibility and reliability studies. Future research should aim to substantiate these preliminary results by employing larger sample sizes and more robust methods. Additionally, investigating the combined effects of SCS and individualized exercise interventions may yield valuable insights into the synergistic benefits of these treatments. Such research could facilitate the development of more comprehensive and effective treatment protocols for patients with PSPS-T2, ensuring the safe and effective management of chronic spinal pain.

## Data Availability

The original contributions presented in the study are included in the article/supplementary material, further inquiries can be directed to the corresponding author.

## References

[1] Yeo J. Failed back surgery syndrome: terminology, etiology, prevention, evaluation, and management: a narrative review. J Yeungnam Med Sci. (2024) 41(3):166–78. 10.12701/jyms.2024.00339 38853538 PMC11294787

[2] Schug SA Lavand’homme P Barke A Korwisi B Rief W Treede RD The IASP classification of chronic pain for ICD-11: chronic postsurgical or posttraumatic pain. Pain. (2019) 160(1):45–52. 10.1097/j.pain.0000000000001413 30586070

[3] Hwan J Lee JH Song KS Hong JY Joo YS Lee DH Treatment outcomes for patients with failed back surgery. Pain Physician. (2017) 20(1):E29–43. 28072795

[4] van de Minkelis J Peene L Cohen SP Staats P Al-Kaisy A Van Boxem K Persistent spinal pain syndrome type 2. Pain Pract. (2024) 24(7):919–36. 10.1111/papr.13379 38616347

[5] North RB Kidd DH Farrokhi F Piantadosi SA. Spinal cord stimulation versus repeated lumbosacral spine surgery for chronic pain: a randomized controlled trial. Neurosurgery. (2005) 56(1):98–106. 10.1227/01.neu.0000144839.65524.e0 15617591

[6] Kumar K Taylor RS Jacques L Eldabe S Meglio M Molet J Spinal cord stimulation versus conventional medical management for neuropathic pain: a multicentre randomized controlled trial in patients with failed back surgery syndrome. Pain. (2007) 132(1–2):179–88. 10.1016/j.pain.2007.07.028 17845835

[7] Bastiaens F van de Wijgert IH Bronkhorst EM van Roosendaal BKWP van Heteren EPZ Gilligan C Factors predicting clinically relevant pain relief after spinal cord stimulation for chronic low back and/or leg pain: a systematic review with meta-analysis and meta-regression. Neuromodulation. (2024) 27(1):70–82. 10.1016/j.neurom.2023.10.188 38184342

[8] Rajkumar S Venkatraman V Yang LZ Parente B Lee HJ Lad SP. Short-term health care costs of high-frequency spinal cord stimulation for the treatment of postsurgical persistent spinal pain syndrome. Neuromodulation. (2023) 26(7):1450–8. 10.1016/j.neurom.2023.01.016 36872148

[9] Miekisiak G. Failed back surgery syndrome: no longer a surgeon's Defeat—a narrative review. Medicina (Kaunas). (2023) 59(7):1255. 10.3390/medicina59071255 37512066 PMC10384667

[10] Gottfridsson R Varkey E Wolf A Gatzinsky K Liljencrantz J Thörn SE Effects of spinal cord stimulation on pain, physical activity, and self-efficacy among patients with neuropathic pain. Pain Manag. (2026) 16(3):185–99. 10.1080/17581869.2025.2608572 41503863 PMC12962678

[11] Zhang N Li C Smith B Patel M Nguyen P. Recent advances and future directions in spinal cord stimulation for chronic pain: a multidisciplinary perspective. Curr Opin Support Palliat Care. (2025) 19(3):162–74. 10.1097/SPC.0000000000000767 40689618

[12] Moens M Goudman L van de Velde D Godderis L Putman K Callens J Personalised rehabilitation to improve return to work in patients with persistent spinal pain syndrome type II after spinal cord stimulation implantation: study protocol for a 12-month randomized controlled trial (OPERA study). Trials. (2022) 23(1):974. 10.1186/s13063-022-06895-5 36471349 PMC9721015

[13] Vicente-Mampel J Hernandez-Zaballos F Falaguera-Vera FJ Sanchez-Poveda D Jaenada-Carrilero E Huertas-Ramirez B Catastrophizing as a predictor for pain perception and disability among patients undergoing spinal cord stimulation. Medicina (Kaunas). (2025) 61(1):141. 10.3390/medicina61010141 39859123 PMC11766538

[14] Tekmyster G Jonely H Lee DW Myerson J Avery M Moradian M Physical therapy considerations and recommendations for patients following spinal cord stimulator implant surgery. Neuromodulation. (2023) 26(1):260–9. 10.1111/ner.13391 33819381

[15] Esomonu C Hagedorn JM. Teaching points: overview of spinal cord stimulation lead migration. Pain Med. (2021) 22(2):520–2. 10.1093/pm/pnaa328 33155048

[16] Koushik SS Raghavan J Saranathan S Slinchenkova K Viswanath O Shaparin N. Complications of spinal cord stimulators: a comprehensive review. Curr Pain Headache Rep. (2024) 28(1):1–9. 10.1007/s11916-023-01178-3 37855944

[17] Deer TR Russo MA Sayed D Pope JE Grider JS Hagedorn JM The neurostimulation appropriateness consensus committee (NACC): recommendations for the mitigation of complications of neurostimulation. Neuromodulation. (2024) 27(6):977–1007. 10.1016/j.neurom.2024.04.004 38878054

[18] Vicente-Mampel J Falaguera-Vera F Sánchez-Poveda D Hernández-Zaballos F Martinez-Soler M Blanco-Giménez P Spinal cord stimulation combined with exercise in patients diagnosed with persistent spinal pain syndrome: study protocol for a randomized controlled trial. PLoS One. (2024) 19(10):e0309935. 10.1371/journal.pone.0309935 39480792 PMC11527166

[19] Falla D Hodges PW. Individualized exercise interventions for spinal pain. Exerc Sport Sci Rev. (2017) 45(2):105–15. 10.1249/JES.0000000000000103 28092298

[20] da Cunha PHM de Andrade DC. The deep and the deeper: spinal cord and deep brain stimulation for neuropathic pain. La Presse Med. (2024) 53(2):104231. 10.1016/j.lpm.2024.104231 38636785

[21] Provenzano DA Heller JA Hanes MC. Current perspectives on neurostimulation for the management of chronic low back pain: a narrative review. J Pain Res. (2021) 14:463–79. 10.2147/JPR.S249580 33628045 PMC7899039

[22] Landelle C Lungu O Vahdat S Kavounoudias A Marchand-Pauvert V De Leener B Investigating the human spinal sensorimotor pathways through functional magnetic resonance imaging. Neuroimage. (2021) 245:118684. 10.1016/j.neuroimage.2021.118684 34732324

[23] West T ElSaban M Hussain N Schappell J Rogers K Orhurhu V Incidence of lead migration with loss of efficacy or paresthesia coverage after spinal cord stimulator implantation: systematic review and proportional meta-analysis. Neuromodulation. (2023) 26(5):917–27. 10.1016/j.neurom.2023.03.016 37204361

[24] Osborne MD Ghazi SM Palmer SC Boone KM Sletten CD Nottmeier EW. Spinal cord stimulator trial lead migration study. Pain Med. (2011) 12(2):204–8. 10.1111/j.1526-4637.2010.01019.x 21143759

[25] Naiditch N Billot M Moens M Goudman L Cornet P Le Breton D Persistent spinal pain syndrome type 2 (PSPS-T2), a social pain? Advocacy for a social gradient of health approach to chronic pain. J Clin Med. (2021) 10(13):2817. 10.3390/jcm10132817 34202362 PMC8269084

[26] Norte G Rush J Sherman D. Arthrogenic muscle inhibition: best evidence, mechanisms, and theory for treating the unseen in clinical rehabilitation. J Sport Rehabil. (2022) 31(6):717–35. 10.1123/jsr.2021-0139 34883466

[27] Nie C Chen K Chen J Zhu Y Jiang J Jin X Altered central pain processing assessed by quantitative sensory testing in patients with failed back surgery syndrome. Neurophysiol Clin. (2022) 52(6):427–35. 10.1016/j.neucli.2022.10.005 36414527

[28] Flynn DM. Chronic musculoskeletal pain: nonpharmacologic, noninvasive treatments. Am Fam Physician. (2020) 102(8):465–77. 33064421

[29] Hutting N Caneiro JP Ong’wen OM Miciak M Roberts L. Patient-centered care in musculoskeletal practice: key elements to support clinicians to focus on the person. Musculoskelet Sci Pract. (2022) 57:102434. 10.1016/j.msksp.2021.102434 34376367

[30] Hutting N Oswald W Staal JB Heerkens YF. Self-management support for people with non-specific low back pain: a qualitative survey among physiotherapists and exercise therapists. Musculoskelet Sci Pract. (2020) 50:102269. 10.1016/j.msksp.2020.102269 33039797

[31] Gibbs MT Last T Marshall P Jones MD. Are the attitudes and beliefs of exercise-based practitioners associated with their use of treatment modalities for people with chronic low back pain? Musculoskeletal Care. (2024) 22(1):e1852. 10.1002/msc.1852 38054520

[32] Barros AMSP Pereira GS da Silva JRT da Silva ML da Costa e Silva MDD Ferrera LMA. The effectiveness of spinal cord stimulation combined with physiotherapy in the management of chronic pain in adults: a systematic review. Front Pain Res (Lausanne). (2025) 6:1620289. 10.3389/fpain.2025.1620289 40766166 PMC12321793

[33] Ganesh GS Khan AR Das S Khan A. Prescription of therapeutic exercise for chronic low back pain management: a narrative review. Bull Fac Phys Ther. (2023) 28(1):47. 10.1186/s43161-023-00156-5

